# Non-immunogenic Induced Pluripotent Stem Cells, a Promising Way Forward for Allogenic Transplantations for Neurological Disorders

**DOI:** 10.3389/fgeed.2020.623717

**Published:** 2021-01-12

**Authors:** Henriette Reventlow Frederiksen, Ulrik Doehn, Pernille Tveden-Nyborg, Kristine K. Freude

**Affiliations:** ^1^Department of Veterinary and Animal Sciences, Faculty of Health and Medical Sciences, University of Copenhagen, Copenhagen, Denmark; ^2^Stem Cell Discovery, Novo Nordisk A/S, Måløv, Denmark

**Keywords:** stem cell treatment, neurological disorder, gene editing (CRISPR-Cas9), iPSC (induced pluripotent stem cell), non-immunogenic, allogenic transplantations

## Abstract

Neurological disorder is a general term used for diseases affecting the function of the brain and nervous system. Those include a broad range of diseases from developmental disorders (e.g., Autism) over injury related disorders (e.g., stroke and brain tumors) to age related neurodegeneration (e.g., Alzheimer's disease), affecting up to 1 billion people worldwide. For most of those disorders, no curative treatment exists leaving symptomatic treatment as the primary mean of alleviation. Human induced pluripotent stem cells (hiPSC) in combination with animal models have been instrumental to foster our understanding of underlying disease mechanisms in the brain. Of specific interest are patient derived hiPSC which allow for targeted gene editing in the cases of known mutations. Such personalized treatment would include (1) acquisition of primary cells from the patient, (2) reprogramming of those into hiPSC via non-integrative methods, (3) corrective intervention via CRISPR-Cas9 gene editing of mutations, (4) quality control to ensure successful correction and absence of off-target effects, and (5) subsequent transplantation of hiPSC or pre-differentiated precursor cells for cell replacement therapies. This would be the ideal scenario but it is time consuming and expensive. Therefore, it would be of great benefit if transplanted hiPSC could be modulated to become invisible to the recipient's immune system, avoiding graft rejection and allowing for allogenic transplantations. This review will focus on the current status of gene editing to generate non-immunogenic hiPSC and how these cells can be used to treat neurological disorders by using cell replacement therapy. By providing an overview of current limitations and challenges in stem cell replacement therapies and the treatment of neurological disorders, this review outlines how gene editing and non-immunogenic hiPSC can contribute and pave the road for new therapeutic advances. Finally, the combination of using non-immunogenic hiPSC and *in vivo* animal modeling will highlight the importance of models with translational value for safety efficacy testing; before embarking on human trials.

## Introduction

Neurological disorders affect over a billion people worldwide (WHO, 2006). Amongst those the most frequent ones are strokes, epilepsy, migraine, Alzheimer's disease (AD) and Parkinson's disease (PD), which have an enormous economic and societal impact as well as diminishing patients' quality of life. At least 12% of all deaths worldwide can be attributed to neurological disorders (WHO, 2006), with the majority still lacking appropriate and curative treatment options. For this reason, attempts of cell replacement therapies have intensified due to their potential to replenish dead and damaged tissues with healthy, and for monogenic neurological disorders, genetically corrected cells.

The idea of replacing damaged or diseased components of our body with healthy ones, has been pursued since the late sixteenth century when the Italian surgeon Gaspare Tagliacozzi was the first to perform a skin transplant (Tomba et al., 2014). He observed that transplants from donor individuals very often resulted in graft rejections. This failure was coined as “The force and power of individuality,” which we nowadays know is the immune system (Siemionow, 2018). Since then, our understanding of the immune system has greatly improved, leading to the development of new strategies for successful transplants. The use of cells for transplantation has become increasingly popular due to their accessibility, and less invasive transplant procedures. Despite improvements, the biggest challenge for a successful transplant still lies in the problem Tagliacozzi encountered 500 years ago, namely our individual immune system limiting comparability e.g., in human leukocyte antigens (HLA) and reducing the chances of finding a matching donor.

If we are to succeed in efficiently applying transplants and cell therapy treatments, a different and more effective approach to resolve graft rejection is required. In this regard, the recent advances in precise genome engineering has launched new possibilities of designing cells, as it enables correction of pathogenic mutations and insertions of new genetic information. This narrative review updates the reader on the application of cell replacement in the treatment of neurological disorders, with a focus on PD where techniques are currently most advanced. Furthermore, a presentation and discussion of potential strategies to implement CRISPR-Cas9 for generating non-immunogenic human induced pluripotent stem cells (hiPSC), which have shown promising results compared to the currently applied strategies, will be made.

## Neurological Disorders

The term neurological disorder spans a wide variety of disorders, as it includes all disorders caused by malfunction of the central and/or peripheral nervous system. Most neurological disorders such as stroke, sporadic PD and ALS and do not have a clear genetic background, even though they have genetic risk factors (Klein and Westenberger, 2012; Boehme et al., 2017; Mejzini et al., 2019). Other disorders such as Huntington's disease, familial AD, and muscular dystrophy have well-known pathogenic mutations. Despite their huge differences, the majority of these disorders share one common trait, which is the vulnerability of specific neurons. This vulnerability manifests in symptoms such as seizures, muscle weakness, cognitive decline, and partial to complete paralysis. Even though diverse neurons and cell types are affected in the various disorders, it is generally accepted that common pathological events lead to degeneration and cell death (Chen et al., 2016). For this reason, beneficial effects of similar treatment targets such as improving mitochondrial function (Mattson et al., 2008) and abnormal inflammatory responses (Skaper et al., 2018) should be investigated. Treatments that have shown benefits in more than one neurological disorder include; electrical deep brain stimulation (Kocabicak et al., 2015), anti-inflammatory drugs (Terzi et al., 2018) and anti-epileptic drugs (Bialer, 2012). Pathological commonalities could be exploited to generate treatment options targeting a broad spectrum of neurodegenerative diseases and elucidate early disease hallmarks central to prevent severe and irreversible damage at later disease stages. Those late disease stages are currently the time points at which the majority of treatment is attempted. Amnjiit Podder et al. showed that besides an overlap in symptoms, several neurological disorders including PD, AD, schizophrenia, autism and migraine have overlaps of genes such as BDNF, DRD2, GAD1, GRIN2A, MAOA, and MTHFR, affecting the functionality of dopamine receptors connecting protein-protein interactions network (Podder and Latha, 2017).

Such genetic studies have the potential to help elucidate which pathways and genes are common between various disorders allowing for more generalized treatment. However, more detailed knowledge of the individuality of neurological disorders may provide crucial information of specific variation in response including why a generalized treatment, such as cell replacement therapy, might not show equal efficiency for different neurological disorders.

The search for treatment and a cure for various neurological disorders is limited by the difficulty of studying the human nervous system and the complex interplay between disease cause, pathology and phenotype. Supplementary Table 1 depicts an overview of neurological disorders, all currently with ongoing clinical trials assessing stem cells as treatment. Additionally, the table lists an overview of the specific pathologies, cell type, areas of nervous system affected and current treatment options. To provide an overview the number of current clinical trials (searchable through www.clinicaltrials.gov) is listed together with a reference to the most recent review paper on stem cell treatment for the specific disorder.

## Cell Replacement Therapy

Cell replacement therapy has increased in popularity since it was showed in 1957 that allogenic bone marrow transplants were successful for treating leukemia (Thomas et al., 1957).

Since then, hematopoietic stem cells have been tested as a potential treatment for the majority of neurological disorders (Sun and Kurtzberg, 2018). Especially neurodegenerative diseases such as PD, have made great progress since the first allogenic study in humans successfully injected fetal mesencephalic tissue containing dopaminergic neurons, into the striatum of two PD patients in 1989 (Lindvall et al., 1989).

**Figure 1:**
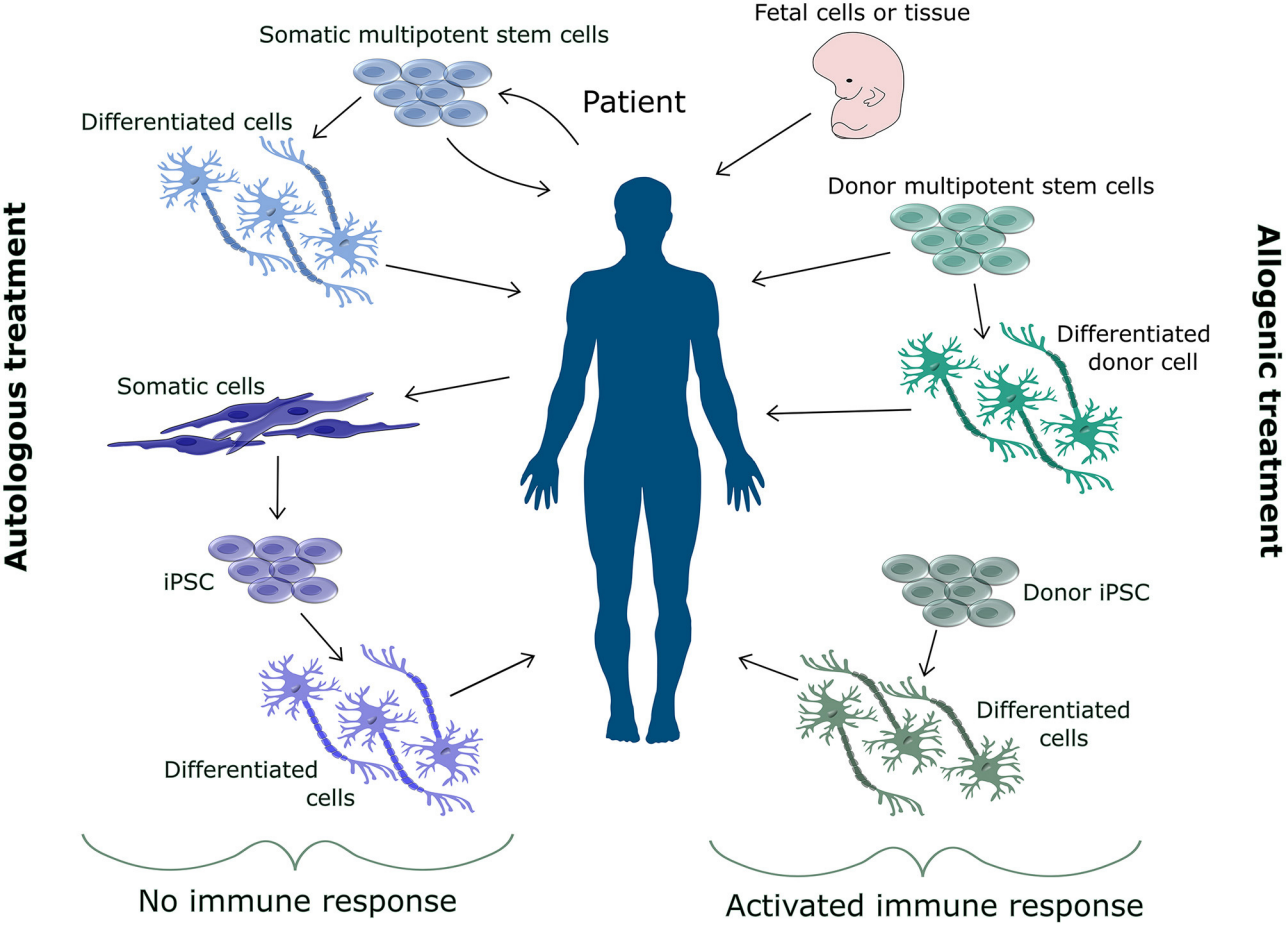
Overview of sources for cell replacement therapy. The source for cell replacement therapy can be either autologous or allogenic. Allogenic cells are associated with an immune response and increase risk of rejection. Therefore, donor cells need to be matched to the patient's immune system. Various allogenic sources such as fetal cells, somatic stem cells, differentiated cells or derivatives from hiPSC can be used for cell replacement therapy. Autologous cells do not cause an immune response. Autologous cells are extracted from the patient either as somatic stem cells or somatic cells for reprogramming. These cells can be injected back into the patient as multipotent stem cells or as differentiated cells.

The great potential of stem cells for treatment lies in the nature of stem cells. Stem cells are able to differentiate into most cell types found in the body and their continuous proliferation capacity allows for large scale treatment. Figure 1 shows the various sources that can be used for stem cell-based treatment, which can be either autologous or allogenic. Allogenic transplants are generally associated with immune response, whilst most autologous transplants cause no immune response (Champlin, 2003). In general pluripotent stem cells are not considered for transplantation, due to their difficult to control proliferation and oncogenic properties (Mamelak et al., 1998; Keene et al., 2009; Mousavinejad et al., 2016). More commonly precursor cells (Strnadel et al., 2018), or multipotent stem cells, such as mesenchymal stem cells (MSC) (Saeedi et al., 2019), are implemented.

Even though autologous transplants are not associated with rejection, studies have shown varying improvements of treatment depending on the disorder. Little improvement has been reported for neurological disorders affecting motor neurons as affected in Amyotrophic lateral sclerosis (ALS) (Goutman et al., 2019) and Spinal Muscular Atrophy (Carrozzi et al., 2012), whereas stabilization and minor improvement is reported in PD (Brazzini et al., 2010; Canesi et al., 2016). Several reasons could underlie those discrepancies in effectiveness such as the source of stem cells and cell type transplanted. For treatment of PD, a substantial difference in results measured by motor scores, non-motor function and cognitive function is seen between transplantation of multipotent stem cells and more differentiated precursor cells. Transplantation of multipotent stem cells gave varying results in progression from no effect (Venkataramana et al., 2010) to stabilization (Canesi et al., 2016) to small improvement (Brazzini et al., 2010). On the contrary transplantation with neuronal precursor cells for dopaminergic neurons have shown consistent improvement in several studies (Piccini et al., 1999; Stoker, 2018). Apart from the various cell type and their sources, an explanation of the different stem cell-based transplantation efficacies, could simply lie within the nature of the disorders. Stem cell-based therapies for disorders such as PD and ALS might be more efficient compared to disorders where multiple cell types are lost such as seen in AD. The difference in efficiency is to some extent caused by the current ability to differentiate certain cell types from stem cells. For PD treatment where dopaminergic neurons are replaced, more than 70 differentiation protocols have been published, resulting in high efficiency (Marton and Ioannidis, 2019). On the contrary, differentiation protocols for motor neurons used for replacement in ALS are not as developed (Gowing and Svendsen, 2011). For treatment of disorders such as AD, transplantation of a single neuron sub type will most likely not be sufficient and co-culture protocols with several cell types still need optimization before being used for stem cell therapy (Goshi et al., 2020).

Allogenic transplants have superior treatment outcomes in disorders such as cancer compared to autologous transplants (Champlin, 2003). It may therefore be likely that allogenic transplants will also be more favorable for stem cell-based treatment of neurological disorders, and allogenic transplants have already been widely used in clinical trials for PD treatment where all showed improvement in “on” and “off” -states and a decrease in Levodopa dose for at least 12 months after treatment (Henderson et al., 1991; Kordower et al., 1995; López-Lozano et al., 1997; Piccini et al., 1999; Brundin, 2000; Venkataramana et al., 2010, 2012). Particularly, autologous and allogenic MSCs (Venkataramana et al., 2010, 2012) have been used as they readily differentiate into neurons (Scuteri et al., 2011) and display protective anti-inflammatory effects on dopaminergic neurons (Kim et al., 2009). A disadvantage of MSCs is the difficulty to grow them *in vitro*, severely hampering the expansion capability and limiting the use of one donor to treat several patients. Another even more popular source for cell mediated PD treatment has been neuronal tissue from aborted fetuses (Henderson et al., 1991; Kordower et al., 1995; López-Lozano et al., 1997; Piccini et al., 1999; Brundin, 2000). This type of treatment possesses a number of disadvantages. Besides the need for immunosuppressive medication, the use of fetal donors presents serious ethical issues. Moreover, the small number of cells available from aborted fetuses is not sufficient to offer generalized treatment.

Cell therapy has a great potential as treatment of a broad variety of neurological disorders, such as the ones listed in Supplementary Table 1, it is of high interest to find the cell type that provides the best platform to initiate personalized treatment. One very promising stem cell type is hiPSCs. They can be generated from various tissues, including nucleated blood cells, which allows easy and pain free access to cellular material. Moreover, hiPSC are widely used to conduct genetic modifications using the CRISPR-Cas9 gene editing tool. If genetic defects can be repaired prior to transplantation in cells which would not be rejected by the host immune system, this would take personalized medicine to an even higher level. Consequently, hiPSC may provide a universal platform for cell therapy especially in combination with gene editing to obtain non-immunogenic cells.

## Immune Rejection

The greatest obstacles for transplantation of hiPSC is the immune response causing graft rejections. The immune response is triggered when the host's immune cells recognize antigens presented by the major histocompatibility complex (MHC) on the surface of the foreign cells as being different to the hosts. This recognition initiates a cascade of signaling pathways releasing cytokines that varies depending on the type of recognition.

There are two classes of MHC. MHCI is expressed on all nucleated cells where they present antigens from the interior of the cell and are required for the activation of CD8+ cytotoxic T-cells (Abbas and Lichtman, 2009). For humans, the MHCI is separated into three major classes called HLA A, B, and C and three minor classes called HLA E, F, and G. The MHCII is expressed on antigen-presenting cells such as dendritic cells where they express antigens from extracellular proteins and are required for activation of CD4+ helper T-cells. The MHCII corresponding HLAs are HLA-DM, HLA-DOA, HLA-DOB, HLA-DP, HLA-DQ, and HLA-DR (Ting and Trowsdale, 2002).

**Figure 2:**
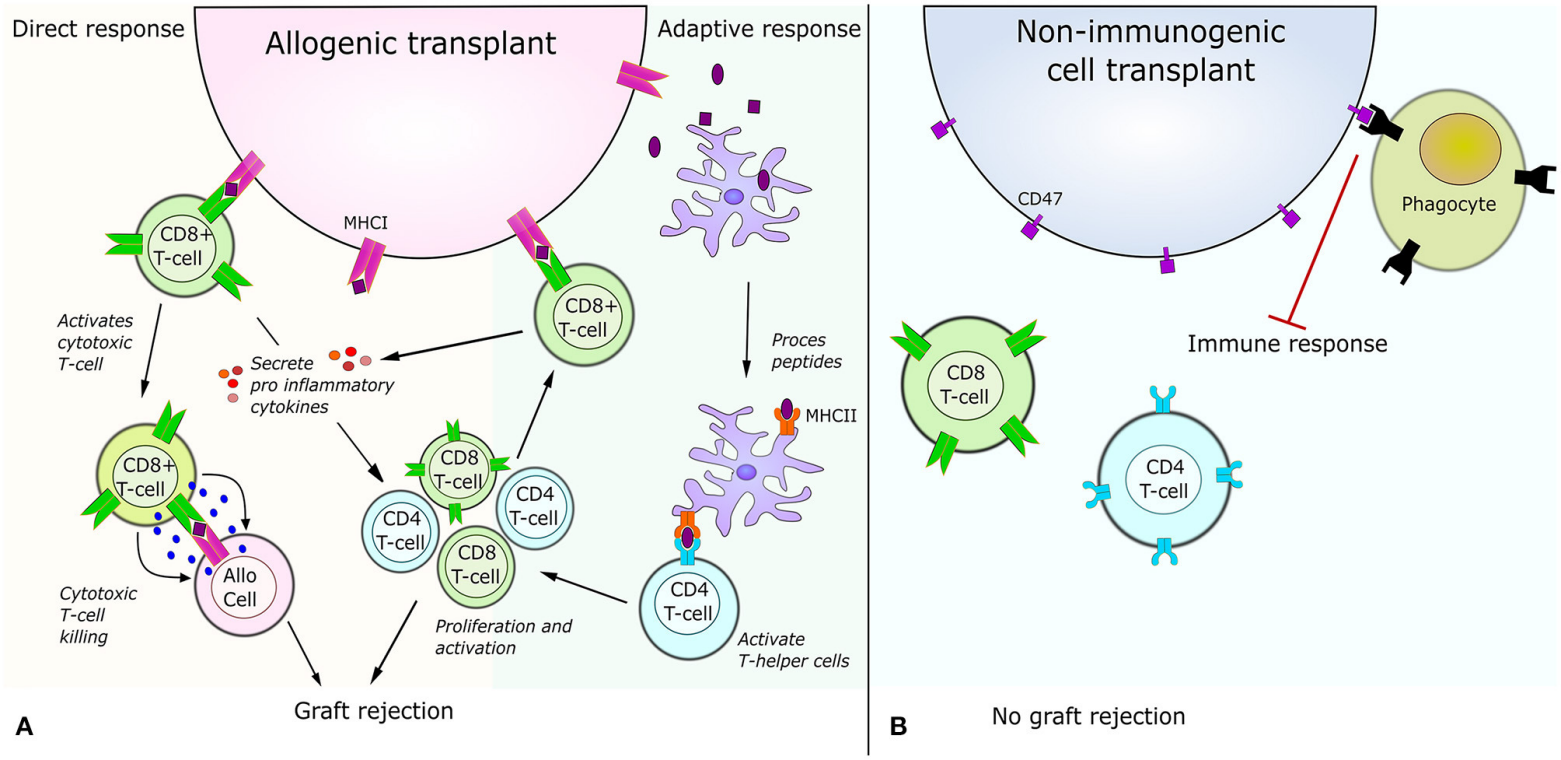
Immune response for allogenic cell transplant vs. non-immunogenic cell transplant. **(A)** Illustration of the immune responses triggered by allogenic transplants. The direct response is caused by CD8 T-cell receptor recognition of a foreign peptide presented by MHCI. This recognition activates the cytotoxic T-cell killing of the allogenic cell and results in secretion of pro-inflammatory molecules amplifying the immune response. The adaptive immune response is caused by antigen presenting cells engulfing peptides from the allogenic cell. After peptides are engulfed, the antigen presenting cell process the peptide and present part of the peptides to helper T-cells via the MHCII. The interaction between helper T-cells and MHCII results in activation and proliferation of CD4 helper T-cells and CD8 T-cells. The activated CD8 T-cells can then recognize peptides presented on the allogenic cells' MHCI and cause a direct response. **(B)** Illustration of the immune response triggered by allogenic transplants with non-immunogenic stem cells. After knock-out of MHCI and MHCII no immune response occurs via the direct or indirect pathway shown to the left. Additionally, over-expression of CD47 on the cell surface acts as a “do not eat me” signal, which further inhibits an immune response.

Immune rejection can generally be divided into two categories depending on whether it is triggered by the immune cells of the host or by the immune cells present in the graft. An immune rejection triggered by the immune cells of the host is caused by host T-cells recognizing MHCI from the recipient or by host CD4+ cells recognizing peptides from the antigen presenting cells of the graft (Figure 2A). Immune rejection can also be caused by the immune cells of the graft recognizing the MHC of their new host (Same response mechanism as Figure 2A). In order to use cell therapy for treating neurological disorders it is necessary to elucidate how the problem of immune rejection can be avoided. Recent approaches to avoid rejection in general include filtering the recipient's blood, such that only regulatory T-cells that aren't able to recognize the antigens of the donor are kept (Sánchez-Fueyo et al., 2020). A different strategy is to desensitize the recipients immune system by removing antibodies and replacing them with antibodies from the donor (Leventhal et al., 2012; Kawai et al., 2014). These approaches have already showed great promise, with several patients being able to stop immune suppressing medication 4–12 month after transplant, even for allogenic donors (Kawai et al., 2008). Other strategies have used quiescent donor dendritic cells, which induced regulatory T-cells, resulting in tolerance of the transplant (Yates et al., 2007) or using co-stimulation blockade of various antigens (Grinnemo et al., 2008). Despite these new approaches, the main strategy to avoid rejection is still the use of HLA matching, as a better match reduces the risk of hyper acute rejection, acute rejection and host vs. graft rejection (Morishima et al., 2002). However, donor HLA matching which is implemented in combination with immunosuppressive drugs can be very challenging for patients with rare HLA types. Furthermore, HLA matching has not been shown to have an effect to prevent chronic rejection (Aron Badin et al., 2019) and graft vs. host disease can still occur if the minor histocompatibility complexes mismatch (Wood et al., 2016).

### Immune Rejection in the Brain

Until recently it was considered easier to perform allogenic transplants in the brain, since the brain was considered immune privileged (Louveau et al., 2015). However, several studies have shown that this is not the case as allogenic brain transplants can cause immune response from neural transplants (Lawrence et al., 1990; Krystkowiak et al., 2007; Fainstein and Ben-Hur, 2018). Other studies show that allogenic transplants do not result in rejection or cause life-threatening or severe symptoms even though an immune response can be measured (Henderson et al., 1991; Kordower et al., 1995; López-Lozano et al., 1997; Venkataramana et al., 2012). For instance a post mortem study of a PD patient receiving transplant of fetal allogenic neurons showed only a mild immune response 4 years after transplantation despite the fact that the patient had only received immune suppressing treatment for 6 months (Mendez et al., 2005). An explanation for this lack of immune rejection has been the low expression profile of MHCI and MHCII in various cell types of the brain such as non-activated microglia and astrocytes (Adelson et al., 2012). A study in non-human primates confirmed low expression of HLA-I in dopaminergic neurons, causing only a mild immune response and no rejection (Morizane et al., 2013). MHCII expression is not only found in microglia, as initially expected, but also in a subpopulation of neural progenitor cells during development (Vagaska et al., 2016). Both MHCI and MHCII are involved in the recognition process of the immune system. A low expression of these is associated with lower immune response as there will be less MHCI and MHCII present at the cell surface to present antigens causing T-cells activation (Figure 2). In this aspect, stem cells display a very low MHCI expression and therefore lower immunogenicity (Drukker et al., 2006). They will however begin to express MHCI and MHCII during differentiation (Lawrence et al., 1990; Liu et al., 2017). Surprisingly, derivatives from stem cells have shown very different results in regards to immune response (Zhao et al., 2015; Wood et al., 2016). Studies in mice with autologous iPSC derivatives show no immune response when injected into the renal space (Guha et al., 2013) the dorsa (De Almeida et al., 2014) or the tail vein (Araki et al., 2013). Another study in a humanized mouse model show varying immune responses depending on iPSC derivative (Zhao et al., 2015). This difference is believed to be partially caused by the minor histocompatibility complexes, which might have different levels of influence depending on the cell type and differentiation state (Goulmy, 1997; Robertson et al., 2007). In general all studies that showed low immune response differentiated the mouse iPSC *in vitro* whereas the other study looked at cell types in a formed teratoma (Zhao et al., 2015).

Even though the majority of treatments, using stem cell derivatives for neurological disorders, only caused mild immune responses, all allogenic stem cell treatments have to be given in combination with immunosuppressive drugs. Stem cell treatment without simultaneous immune suppressive drugs has been shown to cause a life-threatening inflammatory state in a patient and a concise review presents several cases of adverse events (Alderazi et al., 2012; Bauer et al., 2018). Furthermore, treatment with immunosuppressants has the serious disadvantage of significantly increasing the susceptibility too infections, and can cause cell death (Inglese et al., 2004; Rocca et al., 2007). Despite the need for immunosuppression, it is investigated if the treatment can be terminated after a period to avoid some of the negative side effects of lifelong immune suppression. Two different methods have already showed to be successful for renal transplants. One works by “re-setting” the circulating immune cells through a drug targeting white blood cells for destruction, followed by blocking the co-stimulatory pathway to ensure that newly formed blood cells will not recognize the graft (Kirk et al., 2014). Alternatively, blood cells from the donor can be injected into the recipient resulting in chimerism of the immune system, which has been shown to cause no rejection in several patients 18 months after transplantation in an ongoing phase 2 clinical trial (Leventhal et al., 2015). Even though these studies were conducted with renal transplants, they provide evidence of feasibility and underline the great potential for transplants of other cell types into the brain.

## Generating Non-Immunogenic IPSC

### hiPSC

**Figure 3:**
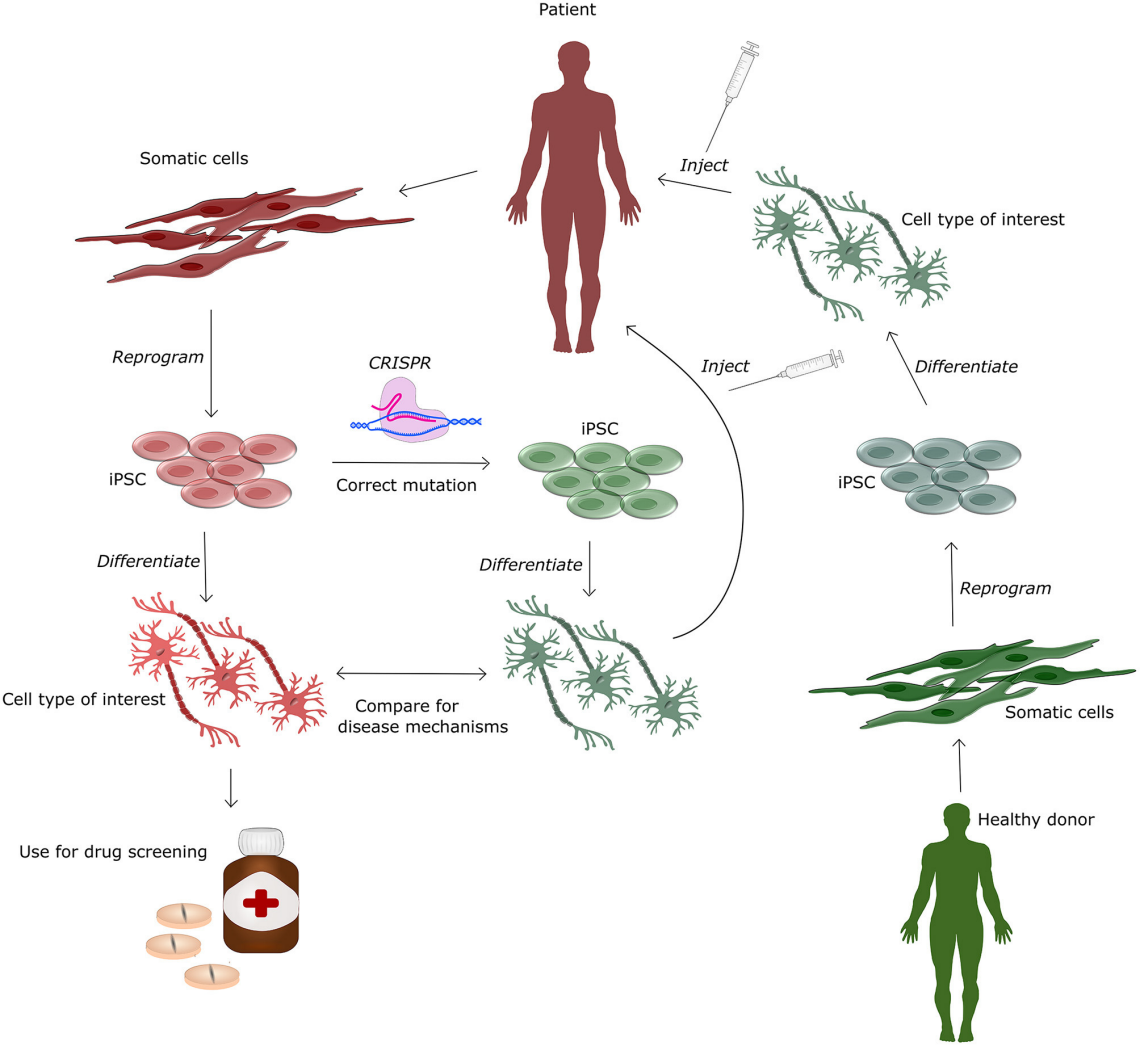
Human induced pluripotent stem cells (hiPSC) for treatment and modeling of genetic diseases. This figure illustrates the various applications of hiPSC, which can be generated from a healthy donor and afterwards differentiated into specific cell types for cell-based therapy or downstream drug screening in *in vitro* based cell systems. The patient derived hiPSC can additionally be genetically corrected using CRISPR-Cas9. This isogenic cell-line can be differentiated and compared to cells from the patient to obtain knowledge of disease and mutation specific mechanisms. Differentiated gene corrected hiPSC can be injected back into the patient for autologous cell therapy.

By developing non-immunogenic hiPSC, one donor can potentially help numerous patients, as a single biopsy reprogrammed into hiPSC can be grown and expanded indefinitely in culture. hiPSCs, are cells that have been reprogrammed from differentiated somatic cells into a pluripotent state, by expressing four transcription factors expressed in the inner cell mass of early blastocysts (Takahashi and Yamanaka, 2006). It was discovered in 2006 by Yamanaka and has since then been widely used in research based on its convenience, availability and reduced ethical constrains compared to embryonic stem cells. The main use for hiPSCs is in the field of research, serving as human *in vitro* disease models, mainly from patients with genetic mutations found in the rare familial forms of AD, PD and ALS (Imaizumi and Okano, 2014) (See Figure 3). Differentiation protocols to generate specific cell types such as glutamatergic and GABAergic neurons, astrocytes, oligodendrocytes and microglia from hiPSC have been greatly improved over the past decade, allowing the investigation of cell type specific disease mechanisms caused by the pathogenic mutations (Ebert et al., 2012). Currently, very few clinical trials are made with hiPSCs, however results from a single clinical trial was published in 2020 showing improvement in one PD patient 24 months post transplantation (Schweitzer et al., 2020). The study injected autologous hiPSC, differentiated into midbrain dopaminergic progenitor cells, into the putamen, left and right hemisphere with 6 months between injections, but as only one subject (age 69 with a 10 year PD history) was included, conclusions are severely limited. Currently, several studies are ongoing, including a collaborative study generating dopamine neurons from HLA-matched donor hiPSC, autologous hiPSC and hESC to treat PD (Barker et al., 2017) and two clinical trials using neural stem cells derived from hiPSC for treatment of PD (trial number: NCT03815071 and NCT02452723). Even though clinical trials with hiPSC have only been conducted in PD patients, preclinical studies with iPSC have shown benefits in mice with spinal cord injury (Cummings et al., 2005), Huntington's disease (An et al., 2012), ALS (Kondo et al., 2014), and stroke (Zhang et al., 2011).

hiPSCs hold further potential for treatment as they can be used efficiently in combination with gene editing tools such as CRISPR-Cas9. This allows to generate isogenic controls for *in vitro* work (Soldner et al., 2011) and to correct pathogenic mutations in patient cells (Pires et al., 2016), hereby providing autogenic cells with a higher therapeutic potential (See Figure 3). Disadvantages of hiPSCs are that the reprogramming procedure may produce minor histocompatibility mismatches causing rejection even for autogenic transplants (Wood et al., 2016) and that reprogramming for up to 50% of all cases are associated with other genetic and epigenetic modifications (Gore et al., 2011). In order to lower the risk of genetic change, non-integrative reprogramming strategies using episomal plasmids, Sendai virus or synthetic mRNA, are favorable for clinical cell lines. hiPSCs that are generated from skin biopsies, may in addition contain unwanted mutations caused by their exposure to UV light. These challenges all have to be overcome before hiPSC can be applied for cell therapy. This underlines the necessity for extensive testing of potential cell lines prior transplantation. Testing should include karyotyping for chromatin validation, validation of differentiation protocol to ensure differentiation potential and whole genome sequencing or targeted sequencing to find unwanted mutations and specific disease genes conferring an external extra risk for other pathologies.

Using a single cell line makes it possible to make extensive quality control, which due to time and money restrains will not be feasible for personalized cell lines (Smith, 2012). Furthermore, using a single cell line for several patients, allows for easier comparison due to the identical genetic background of the transplants. The stem cell lines would have to go through quality control on a regular basis, as *in vitro* culture is known to introduce genomic changes (Peterson and Loring, 2014). Furthermore, the lines should be screened for other genetic risk factors, which could be corrected using CRISPR-Cas9. Correction of genetic risk factor SNPs, or even exchanging them to preventive ones, could result in generation of superior cells for transplantation. Generation of such superior cells falls into a new category of gene editing, posing ethical considerations that must be justified prior to proceeding (Mikkelsen et al., 2019).

### CRISPR-Cas9

CRISPR is the natural adaptive immune system in bacteria (Mojica et al., 2005), a system in which the Cas9 endonuclease targets and cleaves the genome of bacteriophages via matching of base pairs. In 2012 Doudna et al. designed the now popular and widely used CRISPR-Cas9 system, which can target almost any site in the mammalian genome by designing a 20 nucleotide RNA sequence in the guide RNA, complimentary to the DNA target site (Jinek et al., 2012). The guide RNA in complex with Cas9 can only attach to the DNA if the guide sequence is located prior to a protospacer adjacent motif which is present in the human genome on average every 42 bp (How Often Are the PAM Sequences Presented in the Mammalian Genome in Average?, 2020). After attachment Cas9 will make a double stranded cut in the DNA 3bp upstream form the protospacer adjacent motif site allowing for either knock-out (KO) of genes or insertion of new genetic material.

CRISPR-Cas9, facilitated by short RNA guide molecules, has become one of the most used gene editing tools. CRISPR-Cas9 is superior in regards to efficiency and simplicity of design, compared to other, protein-based gene editing tools such as TALENS and ZINC finger nucleases. CRISPR-Cas9 has mainly been used to generate KO of various genes, which has an efficiency of up to 100% for hiPSC (Li et al., 2018) and over 80% for human embryonic stem cells (Bohaciakova et al., 2017), varying from cell line to cell line. Another popular application, for CRISPR-Cas9 is to edit genomic information by specific insertions. CRISPR-Cas9 mediated insertions have a much lower efficiency compared to KOs with around 40% efficiency in hiPSC (Xu et al., 2018). This is because the gene editing is dependent on the less apparent repair mechanism: homology directed repair, which relies on a template providing the new genetic information. Insertions of various sizes from 1 bp (Okamoto et al., 2019) to several kbp (He et al., 2016) have been successful, allowing the use of CRISPR for a wide range of studies.

In hiPSC, CRISPR-Cas9 has generally been used to generate cell models to investigate the cellular pathologies of neurological disorders with defined genetic background. In these models the known pathogenic mutation, such as for example the A53T mutation in the SCNA gene in PD cell lines, can be corrected with the healthy nucleotide to obtain a gene-corrected cell line. If the disease phenotype is mutation dependent all cellular disease phenotypes should be absent and thereby recued via CRISPR-Cas9 gene editing (Lee et al., 2018). Another option is to introduce pathogenic mutations into a “healthy” hiPSC lines to generate a cell lines that should show a phenotype similar to the patients cell lines (Frederiksen et al., 2019). These corrections or insertions allow for comparative studies (Zhang et al., 2017) (see Figure 3) and furthermore allow for future opportunities of patient specific therapies (Safari et al., 2020).

Gene editing has not only been applied in disease modeling. Cell therapy, implementing gene editing, has already been tried in rodents, where Adenovirus was used to therapeutically insert the gene tyrosine hydroxylase in MSC with subsequent transplantation into brains of PD mouse models, successfully increasing dopamine levels (Lu et al., 2005). In humans, the potential of using gene editing for treatment has already been shown for other disorders, such as leukemia, where gene editing has been used to save the lives of two infants. This was done by gene editing T-cells to express chimeric antigen receptor against the B cell antigen CD19 (Qasim et al., 2017). Those genetic engineered T-cells seek out CD19+ B cell acute lymphoblastic cancer cells and eliminate them. Those pioneer works underline that gene editing in stem cells has the potential to enhance a particular cell type allowing for more efficient stem cell treatment.

One of the current challenges in using CRISPR-Cas9 for gene editing include off-targets and varying on-target efficiencies, which is lower when using other tools such as TALENs. Off-target effects caused by CRISPR-Cas9 has been highly debated, as one of the greatest problems with CRISPR-Cas9 and several researchers reported genomic changes caused by off-target events (Cho et al., 2014). Variation of on-target efficiencies can be attributed to cell type differences and target sites, which makes it necessary to design and test several guides. One explanation for varying on-target efficiency is the accessibility of the DNA. Heterochromatin is epigenetically modified to be hypermethylated and tightly packed, which is predicted to be less accessible than euchromatin (Janssen et al., 2019). This varying efficiency of gene editing can affect personalized treatment with autologous cells compared to implementing a universal allogenic donor where no gene editing is needed. As previously mentioned those allografts are subject to rejection responses. Rejection responses could be suppressed by generating KOs of various genes encoding structural components of the immune system.

### Current Non-immunogenic Cell Lines

Creation of a non-immunogenic cell line will allow for transplantation to multiple recipients. This will lower the cost of treatment and reduce the time, as cells can be banked and are readily available in clinical settings. The quest for non-immunogenic cells by gene editing is relatively new but it has already led to a multitude of research and various approaches listed in Table 1. Allogenic teratomas, fibroblasts and cardiomyocytes where shown to be protected from rejection by continuous expression of immunomodulatory molecules such as CTLA4-Ig and Programmed death-ligand 1 (PD-L1) (Rong et al., 2014). Even though this approach showed low immunogenicity, most approaches are based on knowledge from cells with reduced immune responses such as stem cells (Liu et al., 2017) and cells at the feto-maternal interface (Tsuda et al., 2019).

**Table 1:** List of studies made to generate non-immunogenic cells.

**Design**	**Method**	**Cell type**	**Year—References**
KO HLA-A, B, and C	CRISPR-Cas9	(HEK) 293T	2017—Hong et al., 2017
KO HLA-A	Zinc finger nuclease	Human T-cells	2013—Torikai et al., 2013
Knock down B2M	shRNA	hiPSC	2016—Börger et al., 2016
KO B2M	CRISPR-Cas9	hiPSC	2018—Bogomiakova et al., 2018
KO B2M	CRISPR-Cas9	hiPSC	2020—Norbnop et al., 2020
KO B2M	TALEN	hESC	2015—Lu et al., 2015
Knock down B2M	siRNA	hESC	2011—Deuse et al., 2011
KO B2M	Targeting vectors	hESC	2015—Wang et al., 2015
KO CIITA	TALEN	hESC	2015—Chen et al., 2015
KO B2M and CIITA	CRISPR-Cas9	hiPSC	2018—Mattapally et al., 2018
KO HLA-A and HLA-B	CRISPR-Cas9	hiPSC	2019—Xu et al., 2019
KO B2M and CIITA, upregulate CD47	CRISPR-Cas9	hiPSC	2019—Deuse et al., 2019

A commonality for these cell types is that they have low or no expression of MHCI, which will decrease immune response (Figure 2) (Yang et al., 2020). For this reason, several researchers have generated cell lines where either the exon encoding various HLAs or the B2M gene has been knocked out. Generating a B2M KO leads to efficient ablation of all HLA-I types, as B2M encodes one structural part of the MHCI complex. Amongst the studies listed in Table 1, the ones knocking out the exon encoding HLA-A, B and C all showed decreased immune response when tested *in vitro* (Torikai et al., 2013; Hong et al., 2017). The studies generating KO or knock down of B2M all implemented guided differentiation of the iPSC or ESC prior transplantation in mice. Neither of these studies showed complete immune rejection or significant increase in immune response. Except for one study, which measured the graft survival after 42 days, all studies made the assessment after a couple of days. Making the assessment short time after transplantation excludes the possibility to consider an immune response mediated by CD4 T-cells and MHCII. The long term assessment of the mice showed a 40% survival of transplanted cells 42 days post injection, which underlines clearly the importance of MHCII for graft recognition and rejection (Deuse et al., 2011).

MHCII is expressed on antigen presenting cells and plays a central role in generating an immune response, justifying why approaches focusing on knocking out only the MHCII in hESC showed no immune response *in vitro* (Chen et al., 2015). By making a KO of MHCII in combination with the MHCI it is possible to generate an efficient immune deficient hiPSC that show no immune response *in vitro*, even after differentiation into cardiomyocytes (Mattapally et al., 2018). It is known that cells lacking the MHCI complex are targets for natural killer (NK) cells which, as expected, is also shown for cells where B2M has been ablated (Sentman et al., 1995; Lu et al., 2015). To avoid cells being targeted by NK-cells, different approaches have been used. One approach has been to keep the HLA-C but make KO of HLA-A, B and the transcriptional coactivator CIITA for MHCII in hiPSC (Xu et al., 2019). This approach showed protection for NK-targeting *in vitro*. The authors argue that by making a cell line only expressing HLA-C, HLA-matching to reduce the risk of rejection becomes a lot simpler. By matching donor and host according to their HLA-C, only 12 cell lines with different HLA-C profiles would be sufficient to serve as donors for 90% of the population (Xu et al., 2019).

A different strategy was pursued by Deuse et al. who had previously found that KO of MHCI in embryonic stem cells was sufficient to decrease the immune response as the embryonic stem cells naturally have a very low MHCII expression profile (Deuse et al., 2011). By investigating the gene expression profiles of cells at the interface of the fetus and mothers blood supply it was found that MHCI and MHCII, as expected, are highly downregulated whereas CD47 is strongly upregulated (Deuse et al., 2019). This led to the design of hiPSCs with CRISPR-Cas9 generated KO of MHCI and MHCII via the B2M and CIITA gene and an upregulation of CD47 by lentiviral transduction to avoid NK-targeting (Figure 2B). The gene edited hiPSC did not cause an immune response even in HLA mismatched allogenic humanized mouse recipients. Data further showed that a lack of immune response persisted upon differentiation of hiPSC into cardiomyocytes and epithelial cells and that both showed long term survival of at least 50 days *in vivo*. The long survival supports the efficiency of the design where all HLA type I and HLA-III were knocked out, except for HLA-G. It has previously been shown that KO of CIITA does not affect the HLA-G expression and furthermore have a preventive effect of NK targeting (Zhao et al., 2014; Mattapally et al., 2018). As both the presence of HLA-G and upregulation of CD47 have preventive effect of NK-targeting, the combination of the two, which is the design used by Deuse et al., may potentially decrease the risk for NK recognition further.

### Risk Management

Regardless of the advantages of non-immunogenic hiPSC, a potential problem is their safety. If the immune system is not able to detect the foreign cells, then the uncontrolled proliferation of stem cells can potentially lead to even more devastating effects. Even though the risk of tumorigenesis is low for hiPSC derived cells, the need for the host to be able to target cells if infected or mutated is still an important aspect of the design. One strategy to increase the safety of non-immunogenic cells is to knock in the HLA-E complex into the B2M locus (Gornalusse et al., 2017). Expression of HLA-E did not cause an immune response and the risk of NK-mediated cell death decreased as HLA-E is involved in NK-cell recognition (Braud et al., 1998). A favorable aspect of this design, is that HLA-E can express peptides from bacteria on the cell surface enabling the host's immune system to recognize the grafted cells in case of an infection (Lampen et al., 2013). Expression of HLA-E however, does not prevent tumor growth (lo Monaco et al., 2011). The main design of non-tumorigenic hiPSC, is by the use of a so-called suicide switch. One such example is the enzyme inducible Caspase-9 (iCaspase9) which is critical to the apoptotic pathway (Wu et al., 2014; Ando et al., 2015). The iCaspase9 transgene has been successfully inserted into hiPSC by lentiviral transfection, and lead to apoptosis within 24 h once induced by chemical stimulation, hereby serving as an inducible “suicide-switch” (Yagyu et al., 2015). iCaspase 9 has furthermore showed to be efficient of inducing apoptosis in both hiPSC derived neurons and astrocytes (Itakura et al., 2017). In 2020, the same design was conducted by using TALENs in both hiPSC and macrophages and showed that the system is efficiently inducing apoptosis in 95-98% of hiPSC and 90% of hiPSC differentiated macrophages (Lipus et al., 2020). A different attempt to generate non-tumorigenic hiPSC has been made by generating a safe cell system inserting a transcriptional link between two genes responsible for cell division (CDK1) and cell suicide (HSV-TK) (Liang et al., 2018). In a similar manner as for the iCaspase9, division and cell survival can be controlled by giving a specific drug ganciclovir which hereby can arrest or/and kill potential tumor formation. A study in 2019 showed risks associated with knock-in of suicide switches (Kimura et al., 2019). The study knocked-in the HSV-TK gene in hiPSC, which is sensitive to ganciclovir. *In vitro*, ganciclovir exposure caused significant cell death, but the same exposure on teratomas *in vivo* showed varying resistance to the drug. The same study also raises doubt whether human safe loci are in fact safe. These findings highlight the need for more research and risk assessments of various non-immunogenic cells designs. To make such risk assessments, a solid and translational model is needed. As the immune system and nervous system are both highly complex systems, *in vitro* modeling lacks the level of detail necessary to get an accurate picture. Most commonly rodents are used for *in vivo* research, but they differ greatly from humans in metabolism, brain structure and immune system (Mestas and Hughes, 2004). Porcine models and non-human primates are highly relevant for studying human diseases, as they both have a long lifespan and great physiological similarities to humans. Even though non-human primates share the greatest similarities to humans genetically, porcine models are preferable in many aspects such as availability, breeding, size, and a 80% overlap of immune parameters (Meurens et al., 2011). Porcine models have already been generated for neurological disorders such as Huntington's disease (Rausova et al., 2017), stroke (Lau et al., 2018), and other neurodegenerative disorders (Perleberg et al., 2018). Such models can provide platforms for testing of non-immunogenic cell lines for treatment prior to human testing.

## Stem Cell Treatment for Neurological Disorders

**Figure 4:**
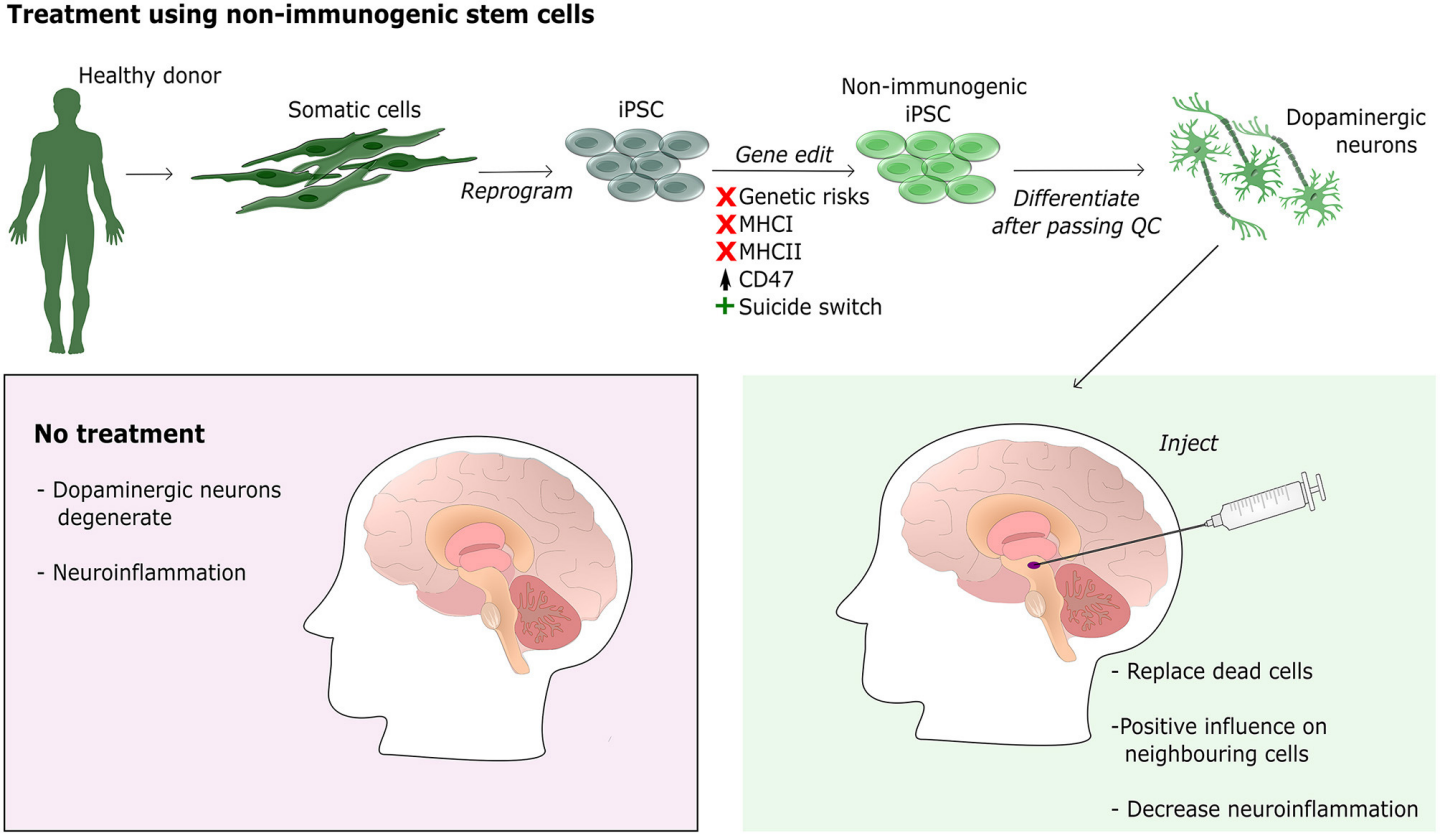
Treatment of PD using non-immunogenic stem cells. The illustration shows the workflow for generating neural progenitor cells for transplantation into the striatum of a PD patient. A healthy donor provides a biopsy, preferably skin, which is then reprogrammed into iPSC using a non-integrative method. iPSC are then gene edited using CRISPR-Cas9 to generate non-immunogenic stem cells by knocking out MHCI, MHCII, and upregulating CD47. Furthermore, a suicide switch is inserted for safety regulation. Once the non-immunogenic cells pass quality control (QC) they can be differentiated into the required cell type, for PD dopaminergic neural progenitor cells. Cells are then injected into the striatum where they can differentiate and integrate. Integrated cells are expected to not only replace dead cells but also to positively influence neighboring cells and decrease neuroinflammation.

The promising results obtained with stem cell-based treatment for PD shows that this form of treatment has great potential in diseases where cells are dying, such as neurodegenerative diseases or in stroke patients. Both diseases have a fundamentally different outcomes, which can lead to profound differences in the success of the engraftment of transplanted cells. In the case of stroke patients, cells would be introduced to the affected site in an otherwise healthy brain environment. For success, the biggest challenge would be graft survival, avoiding tumor formation and functional connection to the existing brain cells. Stem cell-based cell replacement therapies for neurodegenerative diseases face the same hurdles as described for stroke, but are additionally challenged by the transplantation into an environment where pathogenic mechanisms are in place, causing degeneration and apoptosis of brain cells. Inserting healthy cells in this type of “hostile” environment can either have a positive effect as healthy neural progenitor cells secrete neuroprotective factors or the transplanted healthy cells can be negatively affected by the environment and result in graft failure (Kelly et al., 2004; Song et al., 2018; Willis et al., 2020). If the healthy cells are able to positively influence the cells of the host, they can potentially delay or even counteract the pathogenic mechanisms, which would result in improvement for the patient [National Institutes of Health (NIH), 2014]. If the host cells affect the healthy cells, worst case would be no effect of the transplant. However, as most neurodegenerative mechanisms are slow to progress it is perhaps more likely that the transplant would result in temporary improvement until the pathogenic mechanisms affect the healthy cells as well. This is supported by findings showing that addition of astrocytes differentiated from MSC to a Parkinsonian rats have beneficial effects likely through the active secretion of neuroprotective factors (Bahat-Stroomza et al., 2009). Another approach showed that injecting non-differentiated MSC in a rat model of neuropathy resulted in decreased level of pro-inflammatory and an increase in anti-inflammatory proteins, supporting that healthy cells can modulate the inflammatory response of other cells via cell to cell interactions (Siniscalco et al., 2011). The properties of donor cells replacing dead cells and shifting host cells toward an anti-inflammatory state make stem cells particularly interesting for cell replacement therapies, as they can be differentiated into various cell types depending on the disorder that needs treatment. For treatment of PD stem cells have been specifically differentiated into dopaminergic neurons *in vitro* (Arenas et al., 2015). Figure 4 gives an overview of a potential treatment strategy for PD using risk optimized non-immunogenic stem cells differentiated into dopaminergic neurons. It should be noted that differentiation protocols for dopaminergic neurons do not produce pure populations (Kriks et al., 2011; Dell'Anno et al., 2014; Gonzalez et al., 2015; Hallett et al., 2015; Kikuchi et al., 2017; Takahashi, 2017). Research in rodents (Kriks et al., 2011) as well as non-human primates (Hallett et al., 2015) have shown that the success of PD treatment is strongly correlated with the purity level of dopaminergic neurons explaining why differentiation protocols using cell sorting, to obtain a more pure population (Dell'Anno et al., 2014; Hallett et al., 2015; Kikuchi et al., 2017) provides more efficient transplants compared to research without cell sorting (Kriks et al., 2011; Gonzalez et al., 2015; Hallett et al., 2015; Takahashi, 2017). Such findings point out the need for optimization of cell differentiation protocols in order for stem cell treatment to increase its potential.

Interestingly, research has shown upregulation of MHCI in murine dopaminergic neurons upon treatment with IFN-gamma and activated microglia, even though they normally do not express MHCI (Cebrián et al., 2014b). This indicates that MHCI is involved in neuroinflammation and could play an important role in the induced cell death seen in neurodegeneration. By transplanting cells lacking the MHCI, the grafted cells might be less susceptible to induced cell death caused by the pathogenic environment in neurodegenerative diseases. For this reason, non-immunogenic stem cells with MHCI KO might be a better choice for treatment of PD and other neurological disorders causing neuroinflammation, compared to autologous cells.

Despite the huge potential for using risk optimized non-immunogenic iPSC for treatment of neurological disorders, challenges still need consideration. One problem is the variation in recovery and improvement of patients. The variations can be explained by factors such as the amount of tissue transplanted, the age of the donor tissue or injection site, which are factors that can be optimized for better treatment. One factor that cannot be changed, is the disease stage of the patient. Patients early in their disease have showed increased improvement to stem cell therapy compared to patients with advanced disease (Venkataramana et al., 2012). This different response calls for early diagnostics in order for stem cell treatment to be most beneficial.

Another issue for using non-immunogenic iPSC is the consequences of a lack of MHCI and II expression. In rats, MHCI has been shown to not only be expressed in neurons (Needleman et al., 2010), but also to play role in the development of the central nervous system (Cebrián et al., 2014a). Two studies in developing human fetuses have shown expression of MHCI in neurons of the lateral geniculate nucleus and the hippocampus during development, with expression changing as development advances (Zhang et al., 2013a,b). Their findings suggest that MHCI is involved in the maturation of neurons, similar to the finding in rodents. If the MHCI is important for neural maturation, it will most likely be problematic to differentiate stem cells with KO of B2M into mature neurons, needed for treatment of disorders such as PD. It is therefore of high interest to test if full differentiation is possible of non-immunogenic cells such as the ones generated by Deuse et al.

As highlighted in this review non-immunogenic hiPSC derivatives have a large potential to treat a variety of disorders and diseases. However, significant advances are required in order to determine if and to what extent this will be applicable for the various neurological disorder.

## Author Contributions

HRF and KKF conceptualized and wrote the article. HRF generated the figures. All authors edited and approved the final version of the article.

## Conflict of Interest

The authors declare that the research was conducted in the absence of any commercial or financial relationships that could be construed as a potential conflict of interest.
