# Supplementary material for: Non-immunogenic Induced Pluripotent Stem Cells, a Promising Way Forward for Allogenic Transplantations for Neurological Disorders
Source: Front Genome Ed. 2021 Jan 12;2:623717. doi: 10.3389/fgeed.2020.623717 (PMC8525385; doi:10.3389/fgeed.2020.623717)
Supplement: Supplementary file 1 [file Table_1.DOCX]

| **Disorder** | **Phenotype** | **Pathology** | **Forms of disease** | **Cell type and area of nervous system mainly affected** | **Stem cell therapy studies** | **Treatment** |
| --- | --- | --- | --- | --- | --- | --- |
| Parkinson’s Disease (PD) | Shaking palsy and trouble balancing | Formation of Lewy bodies from α-synuclein inhibit the TrkB/TrK pathway resulting in death of dopaminergic neurons. (1) | Genetic form with mutations in PD related genes (alpha-synuclein, Parkin, PINK1, LRRK2, Dj-1, VPS35 and GBA1). Majority of cases are sporadic | Dopaminergic neurons.  Substansia nigra pars compacta (part of midbrain) | Review 2018: (2)  Current clinical trials: 22. | Treatment of symptoms with Levodopa, dopamine agonists or monoamine oxidase-B inhibitors |
| Alzheimer’s disease | Cognitive decline, memory loss and malfunction in speech | Ab-plagues, tau tangles and increased neuroinflammation, causing cell death | Familial form (early onset): mutations in PSEN1^[[1]](#footnote-1)^, PSEN2^[[2]](#footnote-2)^ or APP^[[3]](#footnote-3)^ gene.  Sporadic form (late onset): majority of cases | Neurons. Mostly choligenic neurons. (3)  Initially affects hippocampus. Later migrates to all areas | Review 2019: (4)  Current clinical trials: 10 | Treatment of symptoms with cholinesterase inhibitors or memantine. |
| Amytrophic lateral sclerosis | Decrease in muscle size due to loss of function and neurodegeneration. | Malfunctioning upper neurons give spaticity of limbs and weakness. Malfunctioning lower neurons give fasciculation and weakness. | 5-10% of cases are familial with 13 genes identified. | Upper and Lower motor neurons.  Motor cortex and peripheral nerves. | Review 2019:(5)    Current clinical trials: 36 | Riluzole extends survival 3-6 month. Various drugs for treatment of symptoms. |
| Stroke | Paralysis, memory loss, malfunctioning speech | Cell death in a defined area of the brain caused by low blood flow and hereby lack of oxygen. | Ischemic, caused by clotting of blood vessel. Hemorrhagic, caused by rupture of blood vessel and transient ischemic attack, caused by temporarily clotting of the vessel. | All cell types.  Can occur anywhere in the brain | Review 2019: (6)  Current clinical trials: 43 | Treatment varies depending on type and area affected.  Anticoagulant medicine for Ischemic and transient ischemic.  Surgery for Hemorrhagic stroke. |
| Multiple sclerosis | Depending on nerves affected it can cause tremor, numbness, loss of vision, dizziness and fatigue. | Caused by chronic inflammation. Axonal loss seen after inflammation resulting in autoimmunity attacking the myelin sheets. | Relapsing and reemitting: episodes of neurological dysfunction with normal periods inbetween.  Primary-progressive: progressive neurological disability occur from onset.  Secondary progressive: progressive disability occur later in disease. | Myelin sheet of neurons  CNS^[[4]](#footnote-4)^ | Review 2017: (7)  Current clinical trials: 45 | Glatiramer to reduce relapse rates.  Interferon beta and Mitoxantrone to reduce exacerbations and progression.  Intravenous immune globulin to prevent relapse after first demyelinating event. |
| Brain trauma | Dilated pupils, headache, vomiting, loss of consciousness, anxiety, personality change | Physical damage due to strain cause cell damage and death.  Can vary from mild temporarily to severe where cell loss is too severe for recovery. | Brain damage can be caused by stroke, tumor formation, trauma or illness.  Traumatic brain injury: external force.  Acquired brain injury: internal force. | All cell types  Can be anywhere in the brain | Review 2018:(8)  Current clinical trials: 12 | Treatment is various forms of therapy depending on area affected by the trauma. |
| Cerebral palsy | Affect movement, muscle tone and posture. Involuntary movement | Caused by damage in immature brain. | Exact cause unknown. Severity of disease varies. Can be genetic, caused by infection during pregnancy or due to brain trauma in fetus. | Cerebral cortex | Review 2019: (9)  Current clinical trials: 20 | Various forms of therapy, orthopedic surgery and medication for treatment of symptoms. |

***Table 1: overview of neurological disorders treated with stem cell therapy***

1. Janda E, Boi L, Carta AR. Microglial Phagocytosis and Its Regulation: A Therapeutic Target in Parkinson’s Disease? Front Mol Neurosci. 2018;11:144.

2. Sonntag KC, Song B, Lee N, Jung JH, Cha Y, Leblanc P, et al. Pluripotent stem cell-based therapy for Parkinson’s disease: Current status and future prospects. Vol. 168, Progress in Neurobiology. Elsevier Ltd; 2018. p. 1–20.

3. Mufson EJ, Counts SE, Perez SE, Ginsberg SD. Cholinergic system during the progression of Alzheimer’s disease: Therapeutic implications. Vol. 8, Expert Review of Neurotherapeutics. NIH Public Access; 2008. p. 1703–18.

4. Zhang F-Q, Jiang J-L, Zhang J-T, Niu H, Fu X-Q, Zeng L-L. Current status and future prospects of stem cell therapy in Alzheimer’s disease. Neural Regen Res. 2019;15(2):242–50.

5. Goutman SA, Savelieff MG, Sakowski SA, Feldman EL. Stem cell treatments for amyotrophic lateral sclerosis: a critical overview of early phase trials. Expert Opin Investig Drugs. 2019;28(6):525–43.

6. Baker EW, Kinder HA, West FD. Neural stem cell therapy for stroke: A multimechanistic approach to restoring neurological function. Vol. 9, Brain and Behavior. John Wiley and Sons Ltd; 2019.

7. Sarkar P, Rice CM, Scolding NJ. Cell Therapy for Multiple Sclerosis. CNS Drugs. 2017;31(6):453–69.

8. Weston NM, Sun D. The Potential of Stem Cells in Treatment of Traumatic Brain Injury. Curr Neurol Neurosci Rep. 2018;18(1):1.

9. Eggenberger S, Boucard C, Schoeberlein A, Guzman R, Limacher A, Surbek D, et al. Stem cell treatment and cerebral palsy: Systemic review and metaanalysis. World J Stem Cells. 2019;11(10):891–903.

1. Presenilin-1 [↑](#footnote-ref-1)
2. Presenilin-2 [↑](#footnote-ref-2)
3. Amyloid precursor protein [↑](#footnote-ref-3)
4. Central nervous system [↑](#footnote-ref-4)
